# Supplementary material for: Mapping the evolution of fertility support policies in China: A content and instrumental analysis
Source: PLoS One. 2025 Oct 9;20(10):e0332137. doi: 10.1371/journal.pone.0332137 (PMC12510515; doi:10.1371/journal.pone.0332137)
Supplement: S1 Appendix — (ZIP) [file pone.0332137.s001.zip › S1 Appendix. 226 original policy documents/33-国家卫生健康委关于开展全国计划生育优质服务先进单位创建活动的通知(附 全国计划生育优质服务先进单位创建活动管理办法(2020年版))(FBM-CLI-4-345663).doc]

国家卫生健康委关于开展全国计划生育优质服务先进单位创建活动的通知(附：全国计划生育优质服务先进单位创建活动管理办法(2020年版))

**发布部门：** [国家卫生健康委员会](https://www.pkulaw.com/law/chl?Aggs.IssueDepartment=60321) [机构沿革](javascript:void(0))

**发文字号：**国卫人口函〔2020〕268号

**发布日期：**2020.07.03

**实施日期：**2020.07.03

**时效性：** [现行有效](https://www.pkulaw.com/law/chl?Aggs.TimelinessDic=01)

**效力级别：** [部门规范性文件](https://www.pkulaw.com/law/chl?Aggs.EffectivenessDic=XE0302)

**法规类别：** [计划生育管理](https://www.pkulaw.com/law/chl?Aggs.Category=09705)

国家卫生健康委关于开展全国计划生育优质服务先进单位创建活动的通知
（国卫人口函〔2020〕268号）

各省、自治区、直辖市和新疆生产建设兵团卫生健康委：
　　为落实《[中共中央 国务院关于实施全面两孩政策改革完善计划生育服务管理的决定](https://www.pkulaw.com/chl/8564756bae805261bdfb.html)》和《[国务院办公厅关于促进3岁以下婴幼儿照护服务发展的指导意见](https://www.pkulaw.com/chl/434b24b8b5769383bdfb.html)》，优化生育政策，促进人口均衡发展，我委决定开展新一轮全国计划生育优质服务先进单位创建活动。现将有关事项通知如下：
　　**一、**总体目标
　　以习近平新时代中国特色社会主义思想为指导，认真贯彻党的十九大和十九届二中、三中、四中全会精神，以加强人口监测预警、促进生育政策和相关经济社会政策配套衔接、大力发展普惠婴幼儿照护服务、完善计划生育家庭扶助关怀制度为重点，在全国评选一批2018年以来工作突出的县（市、区）。通过全国计划生育优质服务先进单位创建活动，发挥先进单位的示范引领作用，推动各地把人口监测与家庭发展工作做好做细做实，持续改善管理，提升服务能力，不断增强群众获得感和幸福感。

　　**二、**主要内容
　　（一） 落实党政责任。党委政府高度重视人口与家庭发展工作，坚持计划生育工作领导小组制度，落实相关部门职责，确保体系健全、责任明确、措施有力、投入到位。把加强人口监测预警、促进生育政策和相关经济社会政策配套衔接、大力发展普惠性婴幼儿照护服务、完善计划生育家庭扶助关怀制度等作为重要工作纳入当地经济社会发展规划，采取有效措施推动落实，并取得良好成效。
　　（二）加强人口监测。认真落实人口监测统计调查制度，动态核查更新全员人口库信息。人口基础数据覆盖全面、信息准确、更新及时。实现卫生健康系统内部以及相关部门之间人口基础数据定期会商共享。定期开展人口形势分析。积极参与并高质量完成人口与家庭发展调查等工作。
　　（三）完善生育支持体系。落实产假、哺乳假、产休假，鼓励试行育儿假。落实就业、社保、医保等政策，打击就业歧视，依法保障生育人群合法权益。在街道社区加强母婴设施、儿童活动场所等公共服务资源配置，打造生育友好、家庭友好的社会环境。开展新型婚育文化建设，倡导适龄婚育，促进社会性别平等、代际和谐。
　　（四）促进婴幼儿照护服务发展。明确负责发展婴幼儿照护服务管理的机构和人员，并落实工作经费。通过多种形式和手段，为家庭提供普惠可及的育儿指导服务。在新建居住区或老旧居住区改建时，建设与常住人口规模相适应的婴幼儿照护服务设施及配套安全设施。在城乡社区综合服务设施建设中，统筹考虑婴幼儿照护服务设施建设，为婴幼儿照护创造安全、适宜的环境和条件。推广农村和贫困地区婴幼儿早期发展项目。引导社会力量积极参与，规范发展多种形式的托育机构，优先发展普惠托育服务。支持企事业单位利用自有场地和资金，在工作场所为职工提供福利性婴幼儿照护服务。相关部门按照各自职责，加强对婴幼儿照护服务的宣传教育、指导、监督和管理，确保婴幼儿的安全和健康。
　　（五）加大特殊家庭扶助关怀力度。落实“双岗”联系人制度，积极发动专业社工、计生协会会员、志愿者、家庭医生等开展“多对一”结对帮扶。落实特殊家庭成员优先享受家庭医生签约服务，为签约对象提供约定的基本医疗、基本公共卫生和个性化健康管理服务。省、市、县三级卫生健康行政部门要分别确定本级至少一家综合性公立医院或同等条件社会办综合性医院作为向特殊家庭成员提供便利医疗服务的协议医院，落实就医绿色通道制度。特殊家庭成员在协议医院就诊时，可持相关身份证件，由医务社工、志愿者或其他人员协助办理挂号、就诊、转诊、取药、收费等手续，必要时提供就医引导、陪诊等便利服务。深入开展“暖心行动”，在街道社区普遍建立“暖心家园”，以精神慰藉和心理疏导为重点，开展形式多样的社会关怀活动。
　　（六）巩固基层网络。基层工作网络健全、队伍稳定、保障有力。定期组织开展培训，积极承担人口监测、婴幼儿照护、特殊家庭帮扶等任务，主动参与健康扶贫、爱国卫生、疾病防控、卫生监督、健康教育等工作。各级计生协会组织完善，六项重点任务落实有力。

　　**三、**工作要求
　　（一）各省级卫生健康委要高度重视，结合实际制定创建活动方案，明确目标任务，细化创建内容和标准，严格评审程序，建立激励机制，强化保障措施。
　　（二）各省级卫生健康委要加强指导，鼓励基层参与，把本周期内务实创新、成效明显、群众满意的县（市、区）评选出来，总结宣传易复制、可推广的先进典型。要严明工作纪律，对弄虚作假等行为，一经查实严肃处理。我委将视情组织专题调研。
　　（三）各省级卫生健康委依据本次评选内容（重点指标附后）评选出自2018年以来的计划生育优质服务先进单位。2020年8月10日前将创建活动方案报送我委，10月10日前将拟命名的先进单位名单及创建活动报告报送我委，年底前我委将命名全国计划生育优质服务先进单位。
　　附件：1.全国计划生育优质服务先进单位创建活动管理办法（2020年版）
　　2.全国计划生育优质服务先进单位创建活动评估重点（2020年版）

国家卫生健康委
2020年7月3日

　　附件1

全国计划生育优质服务先进单位创建活动管理办法（2020年版）

　　**第一条**　根据《[中共中央 国务院关于实施全面两孩政策改革完善计划生育服务管理的决定](https://www.pkulaw.com/chl/8564756bae805261bdfb.html)》和党的十九大、十九届二中、三中、四中全会精神，为规范全国计划生育优质服务先进单位创建活动，制定本办法。

　　**第二条**　全国计划生育优质服务先进单位是国家卫生健康委授予的在人口监测和家庭发展工作中重点突出、成效明显、群众满意，能够发挥示范引领作用的县（市、区）的荣誉称号。

　　**第三条**　全国计划生育优质服务先进单位创建活动以县（市、区）为单位开展，3年为一评选周期，期满后荣誉称号自动终止。

　　**第四条**　全国计划生育优质服务先进单位名额实行总量控制，每一周期各省（区、市）和新疆生产建设兵团评选出的计划生育优质服务先进单位比例不超过应参评单位总数的15%。

　　**第五条**　创建活动评选坚持以习近平新时代中国特色社会主义思想为指导，贯彻落实中央决策部署，坚持以人为本、分类指导、严守标准、公开公正公平的原则。

　　**第六条**　国家卫生健康委统筹协调和指导创建活动，制定管理办法和评估重点，组织公示和命名表彰。
　　省级卫生健康委组织本地区的创建活动，制定创建活动方案，细化评估内容，负责评审和监督。

　　**第七条**　全国计划生育优质服务先进单位的评选按照县级申报、市级推荐、省级评审、国家命名的程序进行。
　　（一）县级申报。具备申报资格的县（市、区），以县级人民政府名义向设区的市级卫生健康行政部门提交申报报告。
　　（二）市级推荐。设区的市级卫生健康行政部门对提出申请的县（市、区）进行审核，严守标准，好中选优，向省级卫生健康委推荐。
　　（三）省级评审。省级卫生健康委对申报县（市、区）进行评审，确定全国计划生育优质服务先进单位名单，公示后上报国家卫生健康委。
　　（四）国家命名。对各省级卫生健康委报送的名单，国家卫生健康委通过政府网站等渠道公示10个工作日，接受社会评议和监督。对反映的相关问题，及时组织核查。通过公示和核查的，授予称号，发文通报，颁发奖牌。

　　**第八条**　省级卫生健康委结合实际，建立健全相应的激励机制。总结推广先进单位的经验，把创建活动不断引向深入。

　　**第九条**　有下列情形之一的，由省级卫生健康委核实，撤销其称号，并报国家卫生健康委备案。
　　（一）发生重大违法违规案件的；
　　（二）发生重大安全责任事故的；
　　（三）工作水平严重滑坡的；
　　（四）弄虚作假、统计数据严重失实的。
　　被撤销称号的，不得参加下一周期全国计划生育优质服务先进单位的申报和评选。

　　**第十条**　对在创建活动中组织指导不力、搞形式、走过场的，国家卫生健康委将予以通报，并核减评选名额。

　　**第十一条**　本办法由国家卫生健康委负责解释。
　　附件2

全国计划生育优质服务先进单位创建活动评估重点（2020年版）

　　**一、**党政领导
　　坚持计划生育工作领导小组制度，党政领导专题研究重点工作，各部门职责明确。工作经费本级投入足额到位，管理规范。将生育支持、婴幼儿照护服务、计生特殊家庭扶助等列入民生工程。

　　**二、**人口监测
　　
　　落实人口监测统计调查制度，高质量完成人口监测工作任务。建立部门间人口数据会商共享机制。定期开展人口形势分析。全员人口库常住人口覆盖率和主要数据项准确率达到90%以上。

　　**三、**生育支持
　　
　　全面落实产假、陪产假等政策，按规定落实哺乳假、发放生育津贴。督促用人单位为孕期和哺乳期妇女提供灵活的工作时间安排及必要的便利条件。应配置母婴设施的公共场所和用人单位基本建成标准化的母婴设施。

　　**四、**婴幼儿照护服务
　　
　　明确负责发展婴幼儿照护服务管理的机构和人员，落实工作经费、完善配套优惠扶持政策。规范发展多种形式的托育机构，优先发展普惠托育服务。无重大安全事故发生。

　　**五、**扶助保障
　　
　　计生特殊家庭联系人制度、优先便利医疗服务落实率达100%，家庭医生签约服务做到应签尽签。深入开展“暖心行动”，县（市、区）域内至少有一家“暖心家园”，开展特殊家庭常态化帮扶活动。近三年，无集体访、越级访和进京访。

　　**六、**基层网络
　　
　　基层工作网络健全，专兼职人员熟悉业务。保障落实村居计生专干或村居承担计划生育职责的工作人员待遇。定期组织人员培训，积极参与健康扶贫、爱国卫生、疾病防控、卫生监督、健康教育等工作。推进计生协会改革，深化基层群众自治。

© 北大法宝：（[www.pkulaw.com](https://www.pkulaw.com/)) 专业提供法律信息、法学知识和法律软件领域各类解决方案。北大法宝为您提供丰富的参考资料，正式引用法规条文时请与标准文本核对。
欢迎查看所有[产品和服务](https://www.pkulaw.com/helps/2.html)。法宝快讯：[如何快速找到您需要的检索结果？](https://www.pkulaw.com/helps/70.html)[法宝](https://www.pkulaw.com/helps/69.html)V6有何新特色？

扫描二维码阅读原文

**原文链接：**<https://www.pkulaw.com/chl/1b4095517c5b7187bdfb.html>
